# A hybrid machine learning-based method for classifying the Cushing's Syndrome with comorbid adrenocortical lesions

**DOI:** 10.1186/1471-2164-9-S1-S23

**Published:** 2008-03-20

**Authors:** Jack Y Yang, Mary Qu Yang, Zuojie Luo, Yan Ma, Jianling Li, Youping Deng, Xudong Huang

**Affiliations:** 1 Department of Radiology, Brigham and Women's Hospital, Harvard Medical School, Boston, MA 02115, USA; 2 Genomic Functional Analysis Laboratory, National Human Genome Research Institute, National Institutes of Health, U.S. Department of Health and Human Services. Bethesda, MD 20852, USA; 3 Department of Endocrinology, First Affiliated Hospital, Guangxi Medical University, Nanning, Guangxi Province 530021, China; 4 Department of Biological Sciences, University of Southern Mississippi, Hattiesburg, MS 39406, USA

## Abstract

**Background:**

The prognosis for many cancers could be improved dramatically if they could be detected while still at the microscopic disease stage. It follows from a comprehensive statistical analysis that a number of antigens such as hTERT, PCNA and Ki-67 can be considered as cancer markers, while another set of antigens such as P27KIP1 and FHIT are possible markers for normal tissue. Because more than one marker must be considered to obtain a classification of cancer or no cancer, and if cancer, to classify it as malignant, borderline, or benign, we must develop an intelligent decision system that can fullfill such an unmet medical need.

**Results:**

We have developed an intelligent decision system using machine learning techniques and markers to characterize tissue as cancerous, non-cancerous or borderline. The system incorporates learning techniques such as variants of support vector machines, neural networks, decision trees, self-organizing feature maps (SOFM) and recursive maximum contrast trees (RMCT). These variants and algorithms we have developed, tend to detect microscopic pathological changes based on features derived from gene expression levels and metabolic profiles. We have also used immunohistochemistry techniques to measure the gene expression profiles from a number of antigens such as cyclin E, P27KIP1, FHIT, Ki-67, PCNA, Bax, Bcl-2, P53, Fas, FasL and hTERT in several particular types of neuroendocrine tumors such as pheochromocytomas, paragangliomas, and the adrenocortical carcinomas (ACC), adenomas (ACA), and hyperplasia (ACH) involved with Cushing's syndrome. We provided statistical evidence that higher expression levels of hTERT, PCNA and Ki-67 etc. are associated with a higher risk that the tumors are malignant or borderline as opposed to benign. We also investigated whether higher expression levels of P27KIP1 and FHIT, etc., are associated with a decreased risk of adrenomedullary tumors. While no significant difference was found between cell-arrest antigens such as P27KIP1 for malignant, borderline, and benign tumors, there was a significant difference between expression levels of such antigens in normal adrenal medulla samples and in adrenomedullary tumors.

**Conclusions:**

Our frame work focused on not only different classification schemes and feature selection algorithms, but also ensemble methods such as boosting and bagging in an effort to improve upon the accuracy of the individual classifiers. It is evident that when all sorts of machine learning and statistically learning techniques are combined appropriately into one integrated intelligent medical decision system, the prediction power can be enhanced significantly. This research has many potential applications; it might provide an alternative diagnostic tool and a better understanding of the mechanisms involved in malignant transformation as well as information that is useful for treatment planning and cancer prevention.

## Background

The National Cancer Institute and National Human Genome Research Institute, both part of the NIH and U.S. Department of Health and Human Services, have launched The Cancer Genome Atlas (TCGA) with an overarching goal of understanding the molecular basis of cancer to improve our ability to diagnose, treat and prevent cancer. The perspective of the TCGA project is that “cancer is not a single disease but a collection of diseases that arise from different combinations of genetic changes. Scientists must be able to analyze the genetic material from different tumors and many patients to uncover the tell-tale genetic signatures of different cancer types.” (). Based on the mission of TCGA, we have proposed a further parallel paradigm on cancer: it is not only the genetic changes (i.e. mutations of genes) but changes of gene expressions and regulatory networks that are ultimately responsible for cancer development. Under this parallel paradigm, mutations of genes and un-mutated genes with differential expressions and alternative splicing cause changes in gene regulatory networks (that also cause cancer) when cells are subjected to unusual environments. We consider that the differences between cancer and normal tissue are small in terms of their genotype but perhaps quite larger when one factors in the correlated “biological behaviour phenotypes.” Therefore, our approach focuses on the investigation of differential expressions of genes among normal, benign and cancerous tissues in addition to the genome-wide survey of cancer genetics.

According to the NHGRI-NIH, the cost to sequence genomes will be covered by major insurance policies. Given this, the era of affordable patient-specific medicine based on the full complement of genes is not too far away. However, highly characteristic cancer marker(s) may not always exist in individual patients because, even for the same type of cancer, the genetic mechanisms may be different. The human genome is abundant with alternative splicing; the same gene might have different protein products.

Our novel medical decision system accounts for this variety by using differential gene expression levels. We developed it using Cushing's syndrome as a condition upon which to test pilot our discoveries that challenge today's pathological and histological methods. Once tested, our intelligent medical decision system achieved 92.6% accuracy on three types of Cushing's syndrome, indicating that the joint use of differential gene expressions has enhanced our ability to diagnose diseases. Our long-term strategy is to investigate differential gene expression levels and regulatory pathways that may lead to cancer. The goal of this paper is to introduce a medical decision system as well as the tumor-associated gene expressions that are behind it. These expressions—once expanded upon—will further improve the system and move it beyond the diagnosing of Cushing's syndrome to other types of tumors.

Cushing's syndrome also called hypercortisolism or hyperadrenocorticism is a common endocrine disorder caused by excessive levels of the endogenous corticosteroid hormone cortisol, which is secreted by the adrenal glands which are in turn related to the regulations by the pituitary gland and hypothalamus in the brain. Cushing's syndrome refers to excess cortisol regardless of its etiology. More than two-thirds of cases are related to Cushing's disease, a syndrome characterized by hypercortisolism secondary to excess production of adrenocorticotropin (ACTH) from a pituitary gland adenoma. Roughly one-fourths cases are Cushing's syndrome that is a group of adrenocortical diseases that include tumors of adrenocortical carcinoma (ACC), adrenocortical adenoma (ACA) and adrenocortical hyperplasia (ACH) that all lead to hypercortisolism. The rest of excessive production of ACTH induced by other cancers such as lung cancer and external sources that cause the symptoms of Cushing’s syndrome are rare (less than 10%). Most of those adrenocrtical tumors are benign, however roughly one-quarter may metastasize. The distinctions among Cushing syndrome cases under pathological analysis may not be obvious or not clinically detectable at all, yet the treatments and prognosis are not only different, but also very often determined inappropriately. Cushing's syndrome, therefore, is a complicated disease type, mainly classified as neuroendocrine tumors, that are, in of themselves, generally difficult to identify as potential malignancies based on clinical symptoms and pathological features [1-4]. To conquer such difficulties, we conducted a survey of human genome and tumor genetics and identified several useful (potential) markers such as the expression profiles of cyclin E, P27kip1, FHIT, Bax, Fas, FasL, PCNA, hTERT and Ki-67 for types of Cushing's syndrome. We selected FHIT, PCNA, and Ki-67 because we consider these 3 markers as the most important and easily managed, given our limited experimental supports as illustrated in the following:

Tumor behaviour and growth are considerably influenced by the expressions of two types of genes in the human genome: the cell proliferating genes (for instance, Ki-67 [6,13] and PCNA) and tumor suppressor genes (for example FHIT).

Recently, the protein-coding gene FHIT (fragile histidine triad) has been identified at chromosomal region 3p14.2. While the biological function of the FHIT in the human genome has not been fully characterized yet, it is known that deletion and the degree of deletion in the gene expression level of FHIT are closely associated to the malignancies and prognosis of variety of human tumors [5,6]. Therefore, FHIT is considered a tumor suppressor.

Malignant tumors are showing necrosis and uncontrolled cell proliferation that is related to a nuclear antigen called Ki-67, a nonhistone nucleoprotein in proliferating cell nuclei. This polypeptide accumulates from G1 -phase to mitosis [7-10]. The role of Ki-67 in the human genome has not been identified but Ki-67 antigen-positive cells have given a more accurate indication of proliferating cells compared to that of PCNA (Proliferating Cell Nuclear Antigen) positive cells in many cancers as PCNA is detectable in almost all quiescent cells adjacent to some tumors. Therefore, Ki-67 is a proliferation antigen which is expressed during all phases of the cell cycle except for the resting of cells in G0. The Ki-67 labelling index has prognostic significance in various types of carcinomas, including ACC in Cushing's syndrome. We measure the expression of Ki-67 as a potential malignant tumor marker.

Proliferation cell nuclear antigen (PCNA) was originally identified as an antigen that is expressed in the nuclei of cells during the DNA synthesis phase of the cell cycle. In human genome, PCNA is protein-coding gene product of a kind of ploy-peptide-in-nuclei that acts as processivity for DNA polymerase delta in eukaryotic cells. This protein-coding gene is highly expressed only in proliferating cells. PCNA helps hold DNA polymerase delta (Pol δ) to DNA. PCNA is clamped to DNA through the action of replication factor C (RFC). In human genome, the expression of PCNA is under the control of E2F transcription factor-containing complexes Therefore, the expression and the protein product of PCNA are linked to the cell cycle. In many cases, PCNA can be used to judge malignancies of various tumors and their degrees of proliferation [11-14];. Our immunohistochemical experiments that measure the expression levels of gene-coding proteins Ki-67 and PCNA confirmed their roles in cell cycle regulation and cell proliferation. Since a highly characteristic malignant marker (say 90% accuracy) has not been found in any neuroendocrine tumors, we therefore developed an integrated medical decision machine using a number of associated markers to predict malignancies and to diagnose different adrenocortical diseases, using FHIT, Ki-67 and PCNA as features in the input space.

## Results

### Patients and tumor samples

The tumor samples were from surgical removals of “visible tumors” of patients at the first affiliated hospital of Guanxi Medical University from 1995 to 2005 and were all paraffin embedded and well preserved. All samples were careful determined by all means of pathological and histological analyses. There are 49 confirmed cases of adrenocortical diseases: they are 14 cases of adrenocortical carcinoma (ACC), 26 cases of adrenocortical adenoma (AC A) and 9 cases of adrenocortical hyperplasia (ACH). All cases have been verified by individual patients' medical records. Nineteen of the samples were from male patients (37.5%), and 30 were from female patients (62.5%). The average age and standard deviation of the patients was 35.84±16.18 years. Typical clinical symptoms, signs and laboratory findings of Cushing's syndrome were observed in all cases (Tables 1 and 2). Low-dose dexamethasone suppression test were not inhibited in all cases.

### Measuring protein coding gene expression levels

Although the immunohistochemical measurements of gene express levels of the antigens are not considered as highly quantitative compared to other expensive methods such as DNA microarray, FISH (fluorescent in situ hybridization) and measuring mRNA by in situ hybridizations using cDNA probes via quantitative Reverse Transcriptase Polymerase Chain Reaction (RT-PCR), the immunohistochemistry is very affordable and the results barely affect the performance of our intelligent diagnosis system. We used FHIT rabbit polyclonal antibody (product of Zhongshan Biotechnology, Beijing, China). Ki-67 and PCNA mouse monoclonal antibody kits (ready-to-use products of Maixin Biotechnology Development Co. Fuzhou, China). Immunohistochemical staining was performed using the Superision TM two-step method. In our experiments, we used known positive sections of corresponding tissue samples as positive controls (such as stomach tissue as the positive control for FHIT; gastric cancer as the positive control for PCNA and breast cancer as the positive control for Ki-67). Phosphate-buffered saline (PBS) was used to replace the first antibody and make the “blank” negative control. The HE dyes were used to make the histological control of samples. Our experiments were performed using standard molecular biology procedures to measure the intensities of positive staining and positive rates of the samples by immunohistochemistry: Brown granules in cell nuclei or cytoplasm are considered as positive signals. Specifically, to measure the expression level of FHIT protein in cytoplasm, brown granule in cytoplasm is a positive signal. To measure the levels of expressions of Ki-67 and PCNA proteins in nuclei, brown granules in nuclei are positive signals. The intensities of signals are graded by staining colors: achromatism is marked as 0, light yellow is marked as 1, light brown is marked as 2, and dark brown is marked as 3. Then we compared and determined the graded levels by percentage of positive cells in same type cells: positive cell rate < 5% is marked as 0, positive cell rate between 6%—25% is marked as 1, positive cell rate between 26% -50% is marked as 2, positive cell rate between 51%—75% is marked as 3, positive cell rate > 75% is marked as 4. Then we combined staining intensities and positive cell rates in same type of cells and determine the overall expression level: mark 0 is negative (−), mark 1-4 is weakly positive (+), mark 5-8 is medially positive (+ +), mark 9-12 is strongly positive (+ + +). Because Ki-67 protein is in nuclei, brown granules in nuclei are positive signal. Positive cell rate < 10% is considered negative (−), positive cell rate between 10%—25% is weakly positive (+), positive cell rate between 25%—50% is medially-positive (+ +), positive cell rate > 50% is strongly-positive ( + + +). The determination of those expression levels are also in accordance with [13,15-19]. The measurements are observed and photographed using the Pathological Image Analysis System, DMR+Q550, Germany.

### Results and analysis

Patients' information such as sex, age, side (left or right) or bilateral (if any), diagnosis date, last occurrence; clinical symptoms, abdominal mass, hypertension, central obesity, moon face, buffalo hump, plethoric face, purple striae, hairiness, weakness, decrease in bone content, ECG abnormity and arteriosclerosis, impaired glucose tolerance, infections, oligomenorrhea or amenorrhea, edema, acne, petechia, headache, decrease in bone content, renal calculi, thin skin, bellyache, myoatrophy have all been carefully annotated with the data of laboratory findings such as blood potassium, blood cortisol (8AM, 4PM, 0AM), blood ACTH (8AM, 4PM, 0AM), 24h urinary 17-OH, 24h urinary 17-KS; medical images such as observed tumor size by B-ultrasonic tomography, CT, MRI, PET (if any) and measuring expression levels of protein coding genes by immunohistochemical staining for FHIT, Ki-67, and PCNA in adrenocortical diseases have been recorded and reviewed. All data were then analyzed by a professional statistical software package called SPSS version 11.5. Probability α = 0.05 is considered as statistically significant. We compared measurements by analysis of variance and rank sum test with paired comparisons and chi-square goodness-of-fit test. We designed filters by ordinal logistic regression.

### Expression of FHIT in hypercortisolism of various adrenocortical diseases results and analysis

**Figure 1:**
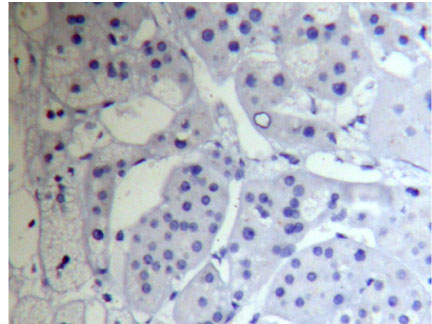
Adrenocortical carcinoma FHIT(−) Superision^TM^ two footworks

**Figure 2:**
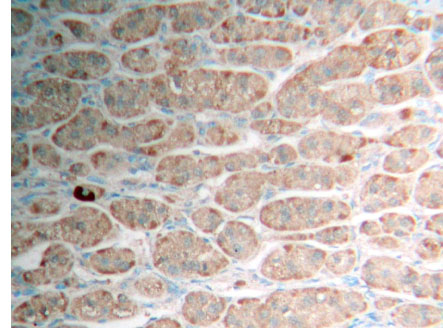
Adrenocortical adenoma FHIT (++) Superision^TM^ two footworks

**Figure 3:**
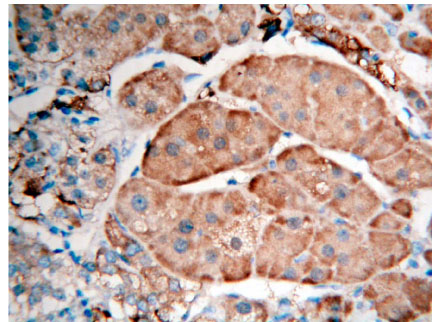
Adrenocortical hyperplasia FHIT ( + + +) Superision^TM^ two footworks

Brown granule of FHIT protein in cytoplasm is considered as positive signal. Among the 14 cases of adrenocortical carcinoma, the weakly positive rate is 42.86% (6/14), both medially positive rate and strongly positive rates are 0% (0/14), total positive rate is 42.86% (6/14); the weakly positive rate in 26 cases of adrenocortical adenomas is 0% (0/26), medially positive rate is 61.54% (16/26), strongly positive rate is 34.62% (9/26), total positive rate is 96.15% (25/26), with only 1 case of negative; in 9 cases of adrenocortical hyperplasia, both weakly positive rate and medially positive rate are 0% (0/9), strongly positive rate is 100% (9/9), total positive rate is 100% (9/9) (Figures 1, 2 and 3). It is evident that the expression level of FHIT decreases while tumor malignancy increases. Statistically analysis showed that the level of total positive rate of adrenocortical carcinoma is significantly lower than both adenoma and hyperplasia (P < 0.0005). There is no statistical difference of the total positive rate between adrenocortical adenoma and hyperplasia (P > 0.05). Comparing the classification of tumors, all cases of adrenocortical hyperplasia are strongly positive; in adrenocortical adenoma, 16 cases are medially positive (61.54%), and 9 cases are strongly positive (34.62%). There are statistically significant differences between them (P < 0.0005). The total positive rate of carcinoma is 42.86%. Six cases of adrenocortical carcinoma are weakly positive, others are negative. Comparing with classification results, there are statistically significant differences between adrenocortical carcinoma and adenoma in negative or weakly positive expression (P < 0.01), as well as between adrenocortical carcinoma and adrenocortical hyperplasia (P < 0.01) (Table 3).

**Table 1:** Laboratory findings of hypercortisolism

Item	$\left(\bar{x}\pm s\right)$
Blood cortisol (nmol/L)	
8AM	846.13±253.995
4PM	748.11±252.344
0AM	633.54±310.857
Blood ACTH (pmol/L)
8AM	5.14±7.08*
4PM	3.99±4.88*
0AM	2.07±4.60*
24h urinary 17-OH (µmol/L)	18.37±11.40*
24h urinary 17-KS (µmol/L)	19.00±8.90*
Blood potassium (nmol/L)	3.48±0.66

PS: data marks * is skew distribution data, shown by median ±quartile

**Table 2:** Expression of FHIT in adrenocortical diseases [n(%)]

**Histology**	**n**	**Negative**	**Positive**			**Total**
		**−**	**+**	**++**	**+++**	**(++++)**
Carcinoma	14	8(57.14)	6(42.86)	0(0.00)	0(0.00)	6(42.86)
Adenoma	26	1(3.85)	0(0.00)	16(61.54)	9(34.62)	25(96.15)
Hyperplasia	9	0(0.00)	0(0.00)	0(0.00)	9(100.00)	9(100.00)

*χ^2^=29.948 P<0.0005

**Table 3:** Expression of Ki-67 in adrenocortical diseases [n(%) ]

**Histology**	**n**	**Negative**	**Positive**			**Total**
		**−**	**+**	**++**	**+++**	**(++++)**

Carcinoma	14	2(14.29)	7(50.00)	4(28.57)	1(7.14)	12(85.71)
Adenoma	26	24(92.31)	2(7.69)	0.(0.00)	0.(0.00)	2(7.69)
Hyperplasia	9	9(100.00)	0.(0.00)	0(0.00)	0.(0.00)	0.(0.00)

### Expression of Ki-67 in hypercortisolism of various adrenocortical diseases

**Figure 4:**
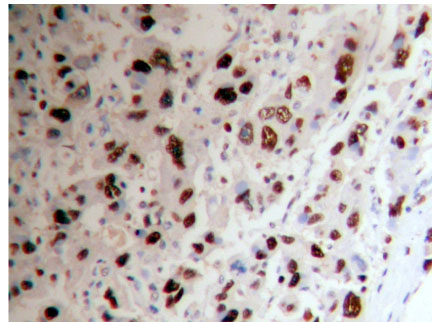
Adrenocortical carcinoma Ki-67 ( + +) Superision^TM^ two footworks

**Figure 5:**
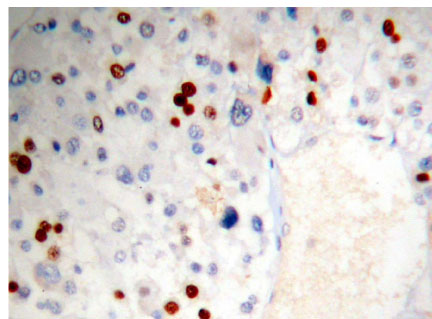
Adrenocortical adenoma Ki-67 (+) Superision^TM^ two footworks

**Figure 6:**
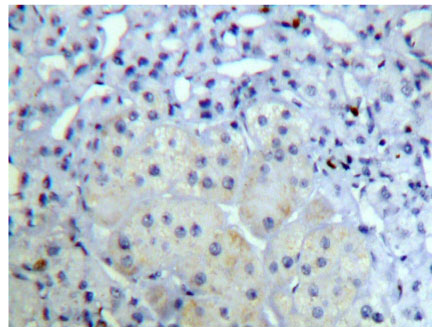
Adrenocortical hyperplasia Ki-67(—) Superision^TM^ two footworks

Ki-67 protein is expressed in cell nuclei. The weakly positive rate in 14 cases of adrenocortical carcinoma is 50% (7/14), medially positive rate is 28.57% (4/14), strongly positive rate is 7.14% (1/14), and total positive rate is 85.71% (12/14). The weakly positive rate in 26 cases of adrenocortical adenoma is 7.69% (2/26), both medially positive rate and strongly positive rates are 0% (0/26), total positive rate is 7.69% (2/26). All the 9 cases of adrenocortical hyperplasia are negative. All of the weakly positive rates, medially positive rates and strongly positive rates are zero (0/9) (Figures 4, 5 and 6). It is evident that the expression level of Ki-67 increases as tumor malignancy increases. Total positive rate of adrenocortical carcinoma is higher than both adenoma and hyperplasia (P < 0.0005). There is no statistically significant difference of the total positive rate between adrenocortical adenoma and hyperplasia (P > 0.05). But compared with the classification of tumors, all adrenocortical hyperplasia cases are negative; in adrenocortical adenoma, 2 cases are weakly positive (7.69%), medially and strongly positive are 0%. There are statistically significant differences between them (P < 0.0005). Compared with classifications of tumors, there is a statistically significant difference between adrenocortical carcinoma and adenoma in medially and strongly positive expression (P < 0.0005), as well as between carcinoma and hyperplasia (P < 0.0005) of medially and strongly positive signals. The expression level in adrenocortical carcinoma is higher than that both in adenoma and hyperplasia (P < 0.0005) (Table 4). In general, we consider high expression level of Ki-67 as a malignant tumor marker. These experimental results indicate that adrenocortical carcinoma can be considered as a malignant cancer. In fact, although adrenocortical carcinomas, generally, carry poor prognoses, still often wrongly considered as benign, the disease is the only occasionally the cause of Cushing's syndrome. Five-year disease-free survival for a complete resection of a Stage I-III ACC (adrenocortical carcinoma) is only approximately 30%. Based on our experimental results, ACC, a common tumor of the adrenal cortex, should be considered at least potentially malignant, while adrenocortical hyperplasia is not only benign but also not considered a tumor but rather an aggregation of unusual cell clusters. ACA should be considered as benign though at risk for malignant transformation.

**Table 4:** Expression of PCNA in adrenocortical diseases [n(%)]

**Histology**	**n Negative**	**Positive**			**Total**
		**−**	**+**	**++**	**+++**	**(++++)**
Carcinoma	14	2(14.29)	7(50.00)	4(28.57)	1(7.14)	12(85.71)
Adenoma	26	1(3.85)	11(42.31)	11(42.31)	3(11.54)	25(96.15)
Hyperplasia	9	2(22.22)	5.(55.56)	2(22.22)	0(0.00)	7(77.78)

* χ^2^=29.948 P<0.0005

### Expression of PCNA in hypercortisolism of various adrenocortical diseases

**Figure 7:**
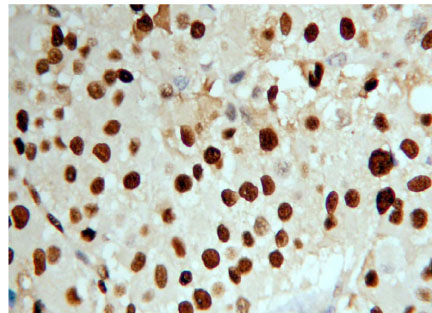
Adrenocortical carcinoma PCNA ( + + +) Superision^TM^ two footworks

**Figure 8:**
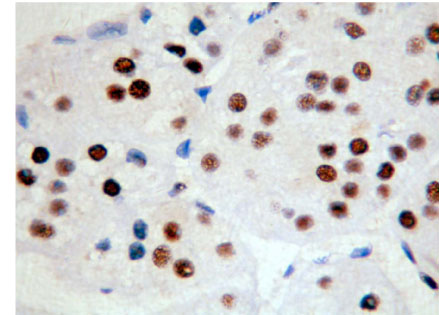
Adrenocortical adenoma PCNA (+ +) Superision^TM^ two footworks

The expression of protein-coding gene PCNA is only in cell nuclei. The weakly positive rate in 14 cases of adrenocortical carcinoma is 7.14% (1/14), medially positive rate is 42.86% (6/14), strongly positive rate is 50% (7/14), and total positive rate is 100% (14/14). The weakly positive rate in 26 cases of adrenocortical adenomas is 42.31% (11/26), medially positive rate is 42.31% (11/26), strongly positive rate is 11.54% (3/26), and total positive rate is 96.15%(25/26). Only one case is negative. The weakly positive rate in 9 cases of adrenocortical hyperplasias is 55.56% (5/9), medially positive rate is 22.22% (2/9), strongly positive rate is 0% (0/9) and total positive rate is 77.78%(7/9). Two cases are negative (Figures 7, 8 add 9). Those experiments indicate that the level of PCNA expression increases as tumor malignancy increases. There is no statistically significant difference in paired comparisons of adrenocortical carcinoma, adenoma and hyperplasia (P > 0.05). Compared with classification of tumors, there is a statistically significant difference between ACC and ACA (P<0.0005), as well as between ACA and ACH (P<0.0005) of medially and strongly positive signals. The expression level in ACA is higher than that in ACC and ACH. Most adrenocortical hyperplasia is negative or weakly positive. Compared with classification results, there is a statistical significance between ACH and ACA (P<0.0005) as well as between ACH and ACC (P<0.0005) of medially and strongly positive signals. The expression level of PCNA in adrenocortical hyperplasia is lower than those in adenoma and carcinoma (Table 5). Those results do not surprise us because we consider Ki-67 as a better malignant marker than PCNA, as PCNA is also detectable in normal tissues adjacent to some tumors. Therefore, it is also plausible that PCNA is detectable in adrenocortical hyperplasia. This also indicated that hyperplasia should not be considered as completely normal tissue rather transforming to benign tumor. PCNA should not be detectable in completely normal tissues. When all statistical analyses are combined into the intelligent diagnostic system, PCNA is actually a little bit better marker than Ki-67 for distinguishing ACC, ACA and ACH. Because most tumors of Cushing's syndrome are benign, nevertheless Ki-67 must be also included into the system to make a reliable diagnosis.

**Table 5:** Expressions of FHIT and Ki-67 in adrenocortical diseases

FHIT	Ki-67			
	(−)	(+)	(++)	(+++)
(−)	0	6	2	1
(+)	2	2	2	0
(++)	16	0	0	0
(+++)	17	1	0	0

Spearman's Correlation Coefficient: rs=-0.718, p<0.0005

### Statistical correlations between the expression levels

Tables 6,7 and 8 show the paired correlations of the expression levels of FHIT, Ki-67 and PCNA in hypercortisolism of adrenocortical diseases (ACC, ACA, ACH). The correlation between the expression levels of FHIT and Ki-67 is negative (r = −0.718, P < 0.0005). The correlation between the expression levels of FHIT and PCNA is negative (r = − 0.449, P = 0.001). The correlation between the expression of Ki-67 and PCNA is positive (r = 0.387, P = 0.006).

**Table 6:** Expressions of FHIT and PCNA in adrenocortical diseases

FHIT	PCNA			
	(−)	(+)	(++)	(+++)
(−)	0	1	5	3
(+)	0	0	2	3
(++)	1	7	7	1
(+++)	2	9	5	2

Spearman's Correlation Coefficient: rs=-0.449, p=0.001

**Table 7:** Expressions of Ki-67 and PCNA in adrenocortical diseases

ki-67	PCNA			
	(−)	(+)	(++)	(+++)
(−)	3	15	12	5
(+)	0	2	5	2
(++)	0	0	2	2
(+++)	0	0	0	1

Spearman's Correlation Coefficient: rs=0.387, p=0.006

Table 9 shows that there is no statistically significant differences among the expression levels of FHIT, Ki-67, PCNA in all 49 cases of adrenocortical diseases with regarding to the clinical parameters including age, sex, side (left or right) (P > 0.05).

**Table 8:** Compare with expression of FHIT, Ki-67, PCNA in adrenocortical diseases and clinical targets

		FHIT		P	Ki-67		P	PCNA		P
Item	n	Total positive	Total rate(%)		Total positive	Total rate(%)		Total positive	Total rate(%)
Age (year)										
<40	30	25	83.33	0.959	9	30	0.511	29	96.67	0.296
≥40	19	15	78.95		14	73.68		17	89.47	
Sex										
Male	17	13	76.47	0.424	7	41.18	0.154	17	100	0.878
Female	32	27	84.38		7	21.88		29	90.63	
Part										
Left	28	22	78.57		9	32.14		26	92.86	
Right	21	18	85.71	0.887	5	23.81	0.815	20	95.24	0.934

**Table 9:** Quantifying of variable

**Variable Targets**		**Quantifying**
Y	Item	Hyperplasia 0, adenoma 1, carcinoma 2

x1	Abdominal mass	have=1, have not=0
x2	Decrease in bone content	have=1, have not=0
x3	F4PM	nmol/L
x4	ACTH8AM	nmol/L
x5	Tumor size	cm3
x6	Metastasis	have=1, have not=0
x7	FHIT	- 0 + 1 ++ 2 +++ 3
x8	Ki-67	**- **0 + 1 ++ 2 +++ 3
x9	PCNA	**- **0 + 1 ++ 2 +++ 3
x10	Purple striae	have=1, have not=0
x11	Urinary 17-KS	µmol/L

Those results indicate tumor markers FHIT, PCNA, Ki-67 should be used jointly in designing an intelligent medical decision system to diagnose the different diseases of Cushing's syndrome as none of the markers is highly characteristic but all are useful.

### Analysis of the related factors of hypercortisolism of various adrenocortical diseases

In order to study the relationships of hypercortisolism and clinical parameters, a number of factors have been screened by ordinal logistic regression. Hypercortisolism of various adrenocortical diseases, including carcinoma, adenoma and hyperplasia, are ordinal multivariate data that can be analyzed by ordinal logistic regression. We choose the pathologic types (i.e. ACC, AC A, ACH) as resulting variable Y. Clinical and laboratory parameters are independent variable X (i.e. FHIT, PCNA, Ki-67). The quantifying of variables is shown by Table 10 (Table 10 only shows the variables that are statistically significant in brief due to limitation of the length of this paper).

**Table 10:** Single factor ordinal logistic regression analysis

**Factors**	**Coefficient of regression**	**Standard error**	**Wald**	**P**
Abdominal mass	3.265	1.172	7.757	0.005
Decrease in bone content	**-**2.165	0.92	5.542	0.019
Blood cortisol 4PM	**-**0.003	0.002	2.981	0.084
Blood ACTH 8AM	**-**0.133	0.59	5.062	0.024
Tumor size	0.002	0.001	4.86	0.027
FHIT	**-**2.904	0.72	16.267	**<**0.001
Ki67	3.262	0.905	12.198	**<**0.001
PCNA	1.912	0.479	15.906	**<**0.001

### Single factor ordinal logistic regression analysis

Among the methods of the ordinal logistic regression, firstly, we performed the single factor ordinal logistic regression analysis because the clinical factors are overwhelmingly diverse but the tumor samples are always limited. We chose the statistically significant level as α < 0.10. Those factors that have statistical significance in single factor ordinal logistic regression analysis are entered as multivariate ordinal logistic regression analysis.

Using the single factor ordinal logistic regression analysis, we found there are no statistically significant differences between a number of factors and diagnosis of hypercortisolism of various adrenocortical diseases (P > 0.10) including but not limited to common clinical information such as sex, age, disease on left or right, last time of disease; clinical symptoms and signs: such as abdominal mass, hypertension, central obesity, moon face, buffalo hump, plethoric face, purple striae, hairiness, weakness, decrease in bone content, ECG abnormity and arteriosclerosis, impaired glucose tolerance, infections, oligomenorrhea or amenorrhea, edema, acne, petechia, headache, decrease in bone content, renal calculi, thin skin, bellyache, myoatrophy; laboratory findings: such as blood potassium, blood cortisol (8AM, 4PM, 0AM), blood ACTH (8AM, 4PM, 0AM), 24h urinary 17-OH, 24h urinary 17-KS; Image findings: such as observed tumor size by B-ultrasonic tomography, CT, MRI. However there are statistically significant differences with regarding to immunohistochemical staining of expression levels of FHIT, Ki-67, PCNA and a few factors among different adrenocortical diseases. This indicates that there are statistically significant differences among the diagnosis of hypercortisolism of various adrenocortical diseases using differential gene expression levels of FHIT, Ki-67, PCNA and a few factors that include abdominal mass, decrease in bone content or fracture, blood cortisol level (4PM), blood ACTH level (8AM), tumor size, and blood cortisol level (4PM, P< 0.10, the others P < 0.05) (table 11). We determined that the above 8 factors are the related factors in diagnosis of hypercortisolism and are used as features of our intelligent medical decision system.

**Table 11:** Single factor ordinal logistic regression analysis

**Factors**	**Coefficient of regression**	**Standard error**	**Wald**	**P**
Constant 1	**-**7.06	2.401	8.646	0.003
Constant 2	1.942	1.733	1.255	0.263
FHIT	**-**3.099	0.891	12.108	0.001
PCNA	2.089	0.752	7.712	0.005

### Multivariate ordinal logistic regression analysis

Those 8 factors that have statistical significances in single factor ordinal logistic regression analysis are entered into multivariate ordinal logistic regression analysis. Factors sifting are adopted into step-by-step method. We entered significance level α = 0.05 and eliminated significance level α = 0.10. We used Chi-Square Goodness-of-Fit test with a result of χ^2^ = 9.422, P = 0.991 > 0.05. Multivariate ordinal logistic regression analysis found that only FHIT and PCNA are strongly related factors of hypercortisolism of various adrenocortical diseases. The correlation between the FHIT and hypercortisolism was negative, the correlation between the PCNA and hypercortisolism was positive (Table 12). Ki-67 is the next useful feature while the rest of the 5 factors are less useful but are not completely useless. This result appears plausible, yet we consider that Ki-67 is a malignant cancer marker. However most of Cushing's syndrome are benign tumors and, as such, it is reasonable that FHIT and PCNA are dominant features to diagnose different types of hypercortisolism in the intelligent machine. Results also indicate that none of the 8 factors is highly characteristic; therefore, we designed an intelligent medical system to enhance the diagnostic accuracy using those 8 factors jointly as illustrated in the following section.

**Table 12:** Accuracies on Our System using Ensemble Methods, Decision Tree and SVM etc. Classifiers on Test Data Set for diagnosis of Cushing's Syndrome of Various Diseases

**Performance**	**Ensemble Methods**	**SOM**	**Decision Trees**	**SVM**
Average Accuracy	92.6%	86.4%	83.3%	81.7%
Standard Deviation	1.8%	2.4%	4.1%	3.6%

## Discussion

The accurate diagnosis of hypercortisolism of various adrenocortical diseases is very critical for suitable treatment planning because effective treatments differ for the various forms of disease associated with Cushing's syndrome. Accurate diagnosis also determines prognosis. Based on our clinical experience, there is no universal effective way to distinguish hypercortisolism among the various adrenocortical diseases. To counter this reality, we used comprehensive information that includes the clinical symptoms and signs, the level of biochemical parameters, hormone tests, medial images, pathologic observation. All of these factors have various limitations and difficulties. Some cases of hypercortisolism involved with adrenocortical diseases are extremely difficult to distinguish based on clinical and pathological analyses. Traditional methods sometime lead to misdiagnosis and wrong choices of the therapeutic schedule [21-25]. This motivated our interest to develop an intelligent medical decision system utilizing tumor associated gene expressions. The system offers a straightforward accurate diagnosis of hypercortisolism of various adrenocortical diseases and, in doing so, represents a realistic and significant clinical diagnostic tool that is highly in demand in today's medicine. We used 8 factors namely FHIT, Ki-67, PCNA, abdominal mass, tumor size, decrease in bone content or fracture, blood cortisol level (4PM), blood ACTH level (8AM); as features in our system for differential diagnosis of hypercortisolism of adrenocortical diseases. FHIH and PCNA are the two most important features for the system, followed by Ki-67. The remaining 5 features are useful.

### Expression level and significance of FHIT

In the human genome, Fragile histidine triad (FHIT) is a gene that was determined and cloned by Ohta et al [26] using Exon acquisition method in 1996. This gene belongs to histidine triad gene families and is the first tumor suppressor gene connected to the fragile site [26] region of 3p14 in human genome. FHIT gene plays a role in cell cycle regulation and apoptosis [27,28]. FHIT gene is expressed in normal human cells. Abnormal expressions of the FHIT gene are connected to diverse forms of malignant tumor development [29-31]. The bioinformatics studies showed that in a great variety of human tumors or tumor cell lines, the FHIT gene presents frequent homozygous deletion, loss of heterozygosity (LOH) and abnormal transcription [32-37]. Furthermore, the bioinformatics studies showed diversiform human epithelial malignancies, FHIT gene absence, abnormal methylation and deplete of FHIT protein express level contribute 70% of human cancers relating the functionality of FHIT. It, thus, can be concluded that FHIT is closely linked to malignant transformation [38]. For many tumors, abnormal FHIT gene regulatory transcription and FHIT protein deletion or re-education have been identified in a great variety of human tumors and tumor cell lines, such as lung cancer, breast cancer, cervical carcinoma, ovarian cancer, and so on [39-60]. It has been detected that the functions of the FHIT gene are associated with tumor development in 50 cases of gastric cancer (Huiping et al [61]) and 84% of them have FHIT gene loss heterozygosity. The FHIT gene reduces carcinogenesis of carcinoma cells. However, the FHIT gene is considered as a carcinoma suppressor gene. We speculate the role of FHIT in inhibiting malignant transformation, [62] however, we will further investigate FHIT in our research to prevent cancer development.

Because FHIT is a protein-coding gene with its ultimate product of fragile histindine triad protein that belongs to the histindine triad protein family with carcinoma suppression activity, FHIT gene mutation leads to FHIT protein abnormal expression. Various carcinogenic factors also lead to abnormal FHIT protein expression, such as reduced levels of FHIT protein expression [64]. FHIT protein deletion and the degrees of deletions in tissues are closely linked to malignancies [5,6] and prognoses [65-68] of tumors have detected pathological analyses.

Reduced expression took place in Stage II -III serous ovarian cancer by Ozaki et al [69] but not in borderline serous ovarian cystadenoma or other histology types of ovarian cancer. It seems that FHIT protein is playing an important role in the malignant course of serous ovarian cancer. The findings of other types tumors also show that FHIT protein deletion or low expression suggests malignant transformation, while on the contrary, high levels of FHIT protein suggest benign status [33-35,37,48-60].

We are the first to systematically measure the expression level of FHIT gene transcript and FHIT protein expression in hypercortisolism of adrenocortical diseases. Found in this study, expression of FHIT in adrenocortical carcinoma is negative or weakly positive, and expression rate is the lowest (P<0.0005). Expression of FHIT in adrenocortical hyperplasia is strongly positive, and expression rate is the highest (P<0.0005). Expression of FHIT in adrenocortical adenoma is between carcinoma and hyperplasia. It suggests that the degree of FHIT gene abnormal transcription and FHIT protein deletion or reduction in adrenocortical carcinoma is more serious than that in adenoma and hyperplasia. FHIT gene abnormal transcript and FHIT protein deletion or reduction are closely linked to the malignancies of tumors in hypercortisolism of adrenocortical diseases. FHIT protein deletion or reduction might indicate malignant transformation.

Found in this study, negative expression cases of adrenocortical carcinoma are 8 (57.14%), and weakly positive expression cases are 6 (42.86%). It suggests that sometimes FHIT protein in malignant carcinoma is not completely deleted but reduced. It is thus that not all adrenocortical carcinoma tissues expressed negative signals but some expressed weakly positive signals. The reason might be linked to tumor malignant degree [65]. Because FHIT gene is a tumor suppressor gene, it can lead cell apoptosis and growth inhabitations of tumors. In some carcinomas with highly malignant degree, FHIT protein is deleted; in the carcinomas with lower malignant degree, FHIT protein not completely deleted but reduced. it appears feasible that we are able to design FHIT microtubule assembles to suppress cell cycle and trigger cell apoptosis in order to suppress tumor development [63] in our future treatment plans.

### Expression level and significance of Ki-67

Ki-67 nuclear antigen is a non-histone nucleoprotein in proliferating cells' nucliues and is closely linked to cell proliferation at region of tenth chromosome [70] in human genome. Ki-67 is associated with tumor malignant degree, tumor infiltrating, metastasis and recurrence. Ki-67 is a tumor associated antigen that has tremendous multiplication capacity and extensive influence on cell proliferation. The function of Ki-67 in human genome has not been identified completely but is linked to cell karyokinesis. Regarded as framework of chromosomes, Ki-67 may be non-histone nucleoprotein matrix inside of chromosomes or around them. It appears that Ki-67 is an important combined characteristic structured protein with little IUP (intrinsic unstructured protein [109], [111], [113]) regions that plays an essential role in keeping the configuration of DNA [71]. Ki-67 is expressed in proliferating cell nucleius at cell cycle stages such as G1 anaphase, S stage, G2 stage and M stage and is expressed in all stages of cell cycle except G0. Because of its half-life is short, Ki-67 degrade speedy when it out of cell cycle, so it become one of the most effective targets of detecting malignant tumor cell proliferation [72]. Results of researches in this field showed that Ki-67 can reflect malignant tumor cell multiplication capacity credibly and speedily; Ki-67 is correlated with a great variety of malignant tumor development, excessive inversion and prognosis [7-10,73-77]. The level of Ki-67 expression is roughly proportional to the degree of malignancy and prognosis [78]. However, we consider Ki-67 is a malignancy marker but is independent to prognosis as reported in [79]. Ki-67 labeling index of ovarian adenocarcinoma indicates significantly higher malignancy than low malignant degree ovarian carcinoma [80]. Ki-67 positive cell percent in high-grade ovarian adenocarcinoma is high, and it is not correlated with tumors histological types, so Ki-67 is useful of ovarian carcinoma's classification but not highly characteristic. Ki-67 can label cells in G1 anaphase, S stage, G2 stage and M stage, but not G0 stage and G1 forepart. It appears that the level of Ki-67 expression can diagnose the malignant tumors but high multiplication capacity while pathological analysis encounters difficulty. Therefore Ki-67 can distinguish benign from malignant tumors. 9 cases of neighboring noncancerous tissue then found that Ki-67 is regarded as a useful antigen for detecting cell multiplication capacity [81].

Ki-67 is closely linked to differential diagnosis in hypercortisolism of adrenocortical diseases [82]. But published reports on it are few. In previous study we researched Ki-67 express in 45 cases of adrenocortical tumors and 9 cases of neighboring noncancerous tissue found that expression of Ki-67 is corrected with adrenocortical tumor. Ki-67 may be taken as one of biomarkers for differentiation of adrenocortical adenomas from adrenocortical carcinoma [84,85]. But in the previous study, the value of Ki-67 for diagnosing adrenocortical carcinoma, adrenocortical adenoma and adrenocortical hyperplasia was not analyzed.

Found in this study, expression rate of Ki-67 in adrenocortical carcinoma is the highest (P<0.0005). It is evident that cell proliferation of adrenocortical carcinomas is more active than adenoma and hyperplasia. Cell proliferation is directly linked to tumor malignancy. Cell proliferation degree consists with tumor malignant degree. So Ki-67 expresses more obviously in adrenocortical carcinoma than in adenoma and hyperplasia (P<0.0005). Because of no abnormal proliferation in normal human tissue cells, Ki-67 is expressed in all human normal cells. Expression of Ki-67 in adrenocortical hyperplasia is not detectable, but in adrenocortical adenoma it is somehow detectable. Because cell proliferation is also observed in benign tumors, even though the expression of Ki-67 in adrenocortical adenoma is low. Along with the cell proliferating degree enhanced, positive grade and positive rate of Ki-67 expression in adrenocortical diseases are enhanced. Because the degree of malignancy is closely linked to tumor cell proliferating degree, Singer and other authors [16,73,85,86] consider Ki-67 to have the ability of tumor infiltrating. Since the expression of Ki-67 increases along with the increasing degree of tumor infiltrating, they consider that Ki-67 might be taken as a parameter to evaluate the ability of tumor infiltrating. Results of this study suggest the expression level of Ki-67 reflects the degree malignancy.

### Expression level and significance of PCNA

Proliferation cell nuclear antigen (PCNA) is a cell cycle protein indispensable to coping of DNA chains. PCNA is only expressed in proliferating cells. Because tumors proliferate faster than normal cells, expression level of PCNA can sensitively reflect the degree of tumor cell proliferation. PCNA is discovered recently as a candidate of tumor marker that reflects cell proliferation degree. It is an antigene specifically expressed in proliferating cell nucleius to measure cells' multiplication capacity [87-90].

PCNA is a nucleoprotein with 36KD molecular weight. It functions as an affiliated protein of DNA polymerase δ. It is indispensable to the copy and rehabilitation of eucaryote DNA main chain and normal cell cycle. Results of researches in this field showed that expression level of PCNA is linked to cell proliferation. PCNA expression in cell nuclieus increases at G1 stage, reaches top at S stage, decrease at G2 stage, but no expression at M and G0 stage. PCNA plays an important role in the adjustment and copy of DNA. PCNA is expressed only in proliferation cells, is expressed much fewer in static cell. The expression level of PCNA can reflect cells' multiplication capacity. PCNA is measurable target for detecting degree of cell proliferation [91].

We consider the degree of cell proliferation degree reflects tumors' biological behaviors; excessive cell proliferation can lead to tumor; cell proliferation is related to tumor excessive invasion and metastasis; therefore tumor cell proliferation can reflect tumor malignancy. PCNA is closely linked to tumor biological behaviors and malignancy [11-14]. High expression level of PCNA reflects high degree of cell proliferation [92,93]. PCNA is related to tumor classification, clinical phase, malignancy, metastasis and prognosis [94]. It is plausible to use PCNA to reflect tumor phase, recurrence and malignancy and classification [95]). It has been reported the detected PCNA expression level in 23 cases of ameloblastoma, indicating that PCNA Index of follicle formation type (34.56%±14.00%) is of higher significance than plexiform type (24.44%±15.74%). It has been reported that detections of Ag-NOR, PCNA and Ki-67 showed no differences in the degree of cell proliferation between follicle formation type and plexiform type [96].

Yanxiaochu [97] et al detected cell proliferating degree in 54 cases of adrenocortical normal tissues, hyperplasia, adenoma and carcinoma by DNA Content, Ag-NOR, PCNA staining, and found that there was no difference between adrenocortical normal tissues and hyperplasia on DNA Content, Ag-NOR, PCNA Index (P>0.05). But there was differences among adrenocortical hyperplasia, adenoma and carcinoma (P<0.01). Our results support and coincident with other findings. Found in this study, expression level of PCNA in adrenocortical carcinoma is the highest, and expression level in adrenocortical hyperplasia is the lowest. The expression level of adrenocortical adenoma is in the middle (P<0.0005). From hyperplasia, adenoma to carcinoma, while the degree of cell proliferating is increasing, the positive rate of PCNA expression is also increasing too. Because the cell proliferating degree between hyperplasia and adenoma is different, expression level of PCNA can be considered as a marker for distinguishing adrenocortical adenoma and adrenocortical hyperplasia.

### The correlations among the expression levels of FHIT, Ki-67 and PCNA

FHIT gene-coding protein is a carcinoma suppressor, it can lead microtubule assembly; however, it can also suppress cell cycle, and may trigger cell apoptosis. In order to suppress tumor proliferation, we must avoid low level of expression of FHIT that limit microtubule assembly and restrain cell apoptosis. Otherwise tumor hyperplasia may overwhelmingly lead to malignancy. Ki-67 can label cells in all stages of cell cycle except G0 as this antigen reflects cell proliferation directly. The expression level of Ki-67 reflects tumor multiplication capacity. The role of Ki-67 is opposite to FHIT. PCNA is mainly expressed in proliferating cell, and is expressed much fewer in static cell. So its expression can also reflect cells' multiplication capacity. The role of PCNA is similar to Ki-67 and is also opposite to FHIT. Found in this study as shown in table 5, 6 and 7, the expression levels of FHIT, Ki-67 and PCNA show distinct patterns in hypercortisolism of various adrenocortical diseases. The correlation between the expression of FHIT and Ki-67 is negative. While the increasing cell proliferating degrees among different diseases of Cushing's syndrome, the expression level of tumor suppressor gene FHIT is reduced, but tumor proliferating antigens Ki-67 and PCN are increased. The correlation between the expression of FHIT and PCNA was negative. The correlation between the expression of Ki-67 and PCNA is positive.

Because FHIT is a tumor suppressor gene, the expression level of FHIT in adrenocortical hyperplasia is high, and in adrenocortical carcinoma is low. On the contrary, expression levels of tumor proliferation cell nuclear antigens Ki-67 and PCNA are roughly proportional to the cell proliferating degree, their levels of expressions in adrenocortical carcinoma are high and in adrenocortical hyperplasia are low.

### Expression levels of FHIT, Ki-67 and PCNA in the diagnosis of hypercortisolism

Found in this study, there are some rules in expression levels of FHIT, Ki-67, and PCNA in hypercortisolism of various adrenocortical diseases. When the expression of FHIT is negative but both of Ki-67 and PCNA are strongly positive, adrenocortical carcinoma is suggested. Or when the expression of FHIT is weakly positive, Ki-67 and PCNA is both strongly positive, adrenocortical carcinoma is suggested, too. When FHIT is strongly positive but both of Ki-67 and PCNA are negative, adrenocortical hyperplasia is suggested. Or when FHIT is strongly positive, Ki-67 is negative, PCNA is weakly positive, and adrenocortical hyperplasia is suggested, too. When FHIT, Ki-67 and PCNA are all positive, adrenocortical adenoma is suggested. The results of this study show that combined detection of the expression of FHIT, Ki-67 and PCNA in hypercortisolism of adrenocortical carcinoma, adenoma and hyperplasia is valuable. They might be applied as credible markers for distinguishing adrenocortical carcinoma, adrenocortical adenoma and adrenocortical hyperplasia. Since those rules are fairly complicated and are difficult for the oncologist who most likely receives only modest training in molecular biology, we need to design an intelligent medical diagnosis system to make a straightforward decision that helps oncologists in their designing of treatment plans.

### The related factors of hypercortisolism of adrenocortical diseases

The related factors of the diagnosis of hypercortisolism of various adrenocortical diseases have not been extensively conducted and the differences of the related factors among adrenocortical carcinoma, adenoma and hyperplasia have not been carefully analyzed up until now. Our research represents the world's first systematic investigation of this type of disease. The values of the related factors in diagnosis of adrenocortical carcinoma have been presented with limit scopes. Lidongxiao [98] et al studied correlation risk factors of 55 cases adrenocortical knub (including non function adenoma, pheochromocytoma, aldosterone producing adenoma, cysts, punctatesubstance hemorrhage, yellow body hemorrhage, metastatic carcinoma and so on, but not including adenoma). They selected 9 factors including age, sex, BMI, knub diameter, knub place, having hypertension or not, having diabetes mellitus or not, having hormone secrete abnormally or not, having other non adrenal tumor, they used statistical analysis to find that knub diameter>2.4cm, mild-abnormal hormone secretion, having hypertension were correlation to the development of adrenal knub. Wubishi [99] et al studied 81 cases hypercortisolism and found that the clinical symptoms and signs such as acne, hairiness, pigmentation, oligomenorrhea or amenorrhea and osteoporosis in adrenocortical hyperplasia (Cushing syndrome) were overwhelmingly outnumbered adrenocortical adenoma. Wangaiping [100] et al studied the diseases using diagnostic values of endocrine laboratory in 70 cases of hypercortisolism. They found that the factors including blood ACTH, blood cortisol, 24hUFC level and large-dose dexamethasone suppression test of inhibition or not were very important to diagnose hypercortisolism diseases. Bornstein [101] et al also found that blood ACTH and large-dose dexamethasone suppression test of inhibition or not played an important role in diagnose hypercortisolism diseases.

In this study 39 factors were investigated including common clinical information such as: sex, age, disease on left or right, last time of disease; clinical symptoms and signs: abdominal mass, hypertension, central obesity, moon face, buffalo hump, plethoric face, purple striae, hairiness, weakness, decrease in bone content, ECG abnormity and arteriosclerosis, impaired glucose tolerance, infections, oligomenorrhea or amenorrhea, edema, acne, petechia, headache, decrease in bone content, renal calculi, thin skin, bellyache, myoatrophy; laboratory findings: blood potassium, blood cortisol (8AM, 4PM, 0AM), blood ACTH (8AM, 4PM, 0AM), 24h urinary 17-OH, 24h urinary 17-KS; medical images: observed tumor size by B-ultrasonic tomography, CT, MRI, PET (if any); Immunohistochemical staining: expression of FHIT, Ki-67, PCNA in adrenocortical diseases. We Analyzed the factors by single factor logistic regression model, and found that 8 factors are likely related to hypercortisolism of adrenocortical diseases including abdominal mass, decrease in bone content or fracture, blood cortisol level (4PM), blood ACTH level (8AM), tumor size, FHIT, Ki-67, PCNA. We also analyzed those factors by multiple factor logistic regression model, and found that the factors of FHIT and PCNA are most valuable. The results suggested that FHIT and PCNA are the more closely related factors for diagnose of hypercortisolism of various adrenocortical diseases. The main reason is the statistical correlation between other factors and these 2 factors are too close. There rest 6 factors are also somehow useful as well.

Clinically, tumor size is often considered as a marker for distinguishing benign or malignant tumor. The diameter of adrenocortical tumor over 5 cm suggests the tendency of malignancy [102]. The place of adrenal glands is deep in abdominal cavity. Too small tumor cannot be touched easily. Touchable abdominal mass means the tumor size is large. Abdominal mass is touchable or not and tumor size contribute to diagnosing hypercortisolism of various adrenocortical diseases. Over half of hypercortisolism patients show decrease in bone content or fracture. Adrenocortical carcinoma patients show more significant decrease in bone content and pathologic fractures are more obviously (still not quite distinct). The reason is likely linked to bone content loss and osteo-anabrosis. But the correlations of decreases in bone content or fracture and tumor malignancy have not been observed at present. Clinically, the hypercortisolism patients' blood cortisol level is often elevated and dysfunction both at 8AM, 4PM and 0AM. The report about differential 4PM cortisol level has not been confirmed because it looks susceptible. In this study, 4PM cortisol level is likely a related factor of hypercortisolism. It suggests dysfunction degree of 4PM cortisol level in adrenocortical carcinoma is more serious than adenoma and hyperplasia. Among hypercortisolism, adrenocortical carcinoma, adenoma and hyperplasia secreting cortisol freely, high blood cortisol level inhibits pituitary secreting ACTH and making blood ACTH level decreased. 8AM blood ACTH level is suggested a likely marker for distinguishing adrenocortical carcinoma, adenoma and hyperplasia.

The high expression level of tumor suppressor gene FHIT may suggest benign tumor. FHIT protein is an accessible target in molecular biology laboratory used to judge various benign tumors. The expression of cell proliferation antigen Ki-67 reflects cell multiplication capacity. Ki-67 can reflect the proliferation rate of malignant tumors. Proliferation cell nuclear antigen PCNA is also an accessible target in molecular biology laboratory to assess the degree of cell proliferation. The expression levels of FHIT, Ki-67 and PCNA in hypercortisolism of various adrenocortical diseases are useful for distinguishing adrenocortical carcinoma, adenoma and hyperplasia but none of them are high characteristic. Therefore this again confirm the needs of an intelligent medical decision system. This study found there are some rules in expression levels of FHIT, Ki-67, and PCNA in hypercortisolism of various adrenocortical diseases. We utilized them jointly in designing an intelligent medical decision system to diagnose hypercortisolism of adrenocortical carcinoma, adenoma and hyperplasia. Because hypercortisolism is a common endocrine disease with increasing occurrence rate recently, development of this medical diagnosis system is important for choosing correct treatment plans and estimating prognosis. The clinical significance of this medical decision system using expression levels of FHIT, Ki-67 and PCNA and 5 related factors is that this system concurred the difficulties of diagnosing hypercortisolism of various adrenocortical diseases.

## Conclusions

The novel intelligent medical diagnose system developed here is originated from a prototype system that we won a novel smart engineering system design award [108]. The new system presented here has significantly enhanced the diagnose of Cushing's syndrome of different diseases that challenges today's medicine, the synergistic effects of the system proved the great effectives of combined artificial intelligence with experimental molecular biology technique. We benchmark our ensemble method against 3 other popular algorithms namely SOM, decision trees C5 and support vector machines SVM-light (table 13). Our intelligent system significantly outperformed those popular machine learning algorithms and exceed 92% accuracy in diagnosis. Along the way, we made several medical discoveries:

1) The expression of FHIT, Ki-67 and PCNA strongly relate to hypercortisolism of different adrenocortical diseases. Expression of FHIT is the highest in adrenocortical hyperplasia, lowest in carcinoma, and middle in adenoma. Expression of Ki-67 and PCNA in adrenocortical carcinoma is the highest, in hyperplasia is the lowest, and in adenoma is the middle. They might be applied as one of markers for distinguishing adrenocortical carcinoma, adenoma and hyperplasia.

2) The expressions of FHIT, Ki-67 and PCNA in hypercortisolism of adrenocortical diseases were paired correlated. The correlation between the expression of FHIT and Ki-67 was negative; the correlation between the expression of FHIT and PCNA was negative; the correlation between the expression of Ki-67 and PCNA was positive.

3) The combined expression of FHIT, Ki-67 and PCNA in hypercortisolism of adrenocortical diseases is valuable. When the expression of FHIT is negative or weakly-positive but both of Ki-67 and PCNA are strongly positive, adrenocortical carcinoma is suggested; when FHIT is strongly positive but both of Ki-67 and PCNA are negative, adrenocortical hyperplasia is suggested; while when FHIT, Ki-67 and PCNA are all positive, adrenocortical adenoma is suggested.

4) It was found by Logistic Regression that 8 factors were likely linked to the diagnosis of hypercortisolism of adrenocortical diseases including FHIT, Ki-67, PCNA, abdominal mass, tumor size, decrease in bone content or fracture, blood cortisol level (4PM), blood ACTH level (8AM) amongst which FHIT and PCNA are the most imprtant features for diagnosis.

The successful development of the world first of its kind intelligent medical diagnosis system marks the beginning of synergistic approaches of artificial intelligence and laboratory molecular biology to diagnose diseases with high accuracy.  The success may predict prognosis and better understanding human genome mechanisms relating to potential malignant transformation.  It also in provides useful information for better treatment planning and cancer prevention.

## Methods

### The intelligent medical diagnostic system

Recently there has been a surge of interest in using ensemble methods to enhance the performance of medical diagnostic systems. Ensemble method is a diverse class of methods that seek to combine the decisions of several (computational intelligence) classifiers in order to improve the performance of the classification task. This class includes:

Consensus networking – In this approach, the test instances are fed into several (computational intelligence) classifiers and majority voting of the classification decisions of these classifiers are taken.

Boosting – This approach is a computational intelligence machine learning meta-algorithm. At each boosting round, a *“weak”* learner is trained with the data and output of the learner is feedback to the learned function, with some strength. Then, the data is re-weighted and boosting is focused on the data that are difficult to learn in the next boosting round, so that future *“weak”* learners will attempt to reduce the mis-classification errors.

Bootstrap Aggregation (“Bagging”) – In this approach, the original data set is sampled (with replacement) to form M “bags” of data, each equal in size to the original dataset; a classifier is constructed based on each of M bags. Then, given an instance to be classified, it can be fed it into each of the M classifiers and take the majority vote of these classifiers to form the final classification decision.

Ensemble methods have been shown to be effective at reducing the generalization error. Several issues arise in the design of such a medical decision system:

• What types of classifiers should be combined? And

• How should they be combined?

As to the first question, our system combines the predictions of decisions from Recursive Maximum Contrast Trees RMCT [106,107], SOFM and Parallel Self-Organzing Hierarchical Neural Networks (PSHNN)[104,105]. As to the second question, we are investigating a multistage classification scheme in which each stage is composed of multiple classifiers whose decisions are combined by majority voting and consensus. Instances that are misclassified by the first stage are passed to the second stage. The idea is: by only focusing on the instances misclassified by the first stage, the second stage can concentrate on the more difficult parts of the feature space and so on. It appears that there is a strong theoretical basis that Boosting with Bagging [112] reduces the variance component of the error under certain conditions and is resistant to over-fitting. This is especially important that we are dealing with a very important but kind of rare type of tumor that is unsuited for a large training sample size (along with all the expensive laboratory measures). We use a variant of ensemble method that is a diverse class of methods that seek to combine the decisions of several computational intelligence classifiers in order to improve the performance of the classification task. Our algorithm is as follows:

• First step:

– Construct two very different computational intelligence classifiers, the variant of the neural network Self-Organizing Feature Map (SOFM) classifier and RMCT.

– Pass the test instance to both classifiers:

- If both classifiers agree, then this is the consensus prediction.

- If they disagree, this may indicate that the instance is difficult to predict reliably, then we use the second step with additions of a third classifier and a more powerful computational intelligence algorithm named Boosting with Bagging to break the tie (we will explain the Boosting with Bagging algorithm in a separate section later on).

• Second step:

– Construct an additional classifier, PSHNN.

Pass the test instance to all 3 classifiers (SOFM, RMCT and PSHNN), but each classifier is also trained by Boosting with Bagging; the consensus prediction is obtained by taking the majority vote of all three classifiers.

A medical decision system or a medical expert system can use Kohonen's SOM. Our development of new variants of neural network based algorithms is Self-organizing Feature Map algorithms (SOFM) and is inspired by the SOM [103] and the PSHNN (Parallel Self-Organizing Hierarchical Neural Networks) algorithms [103,104]. The computational intelligence system we developed here is a machine learning system rather a medical expert system (which is a much more sophisticated system governed by the rules based on the opinions from the experts in a specified field). Though our system is relatively simpler and more straightforward than an expert system, it can actually be more useful and more accurate for a well-defined highly specific task because all features are the solid experimentally measured gene expression and clinical measurement values rather than diverse opinions from human experts or predicted gene expression values from pure bioinformatics software tools.

In the Kohonen's neural networks SOM algorithm, each neuron has associated with a topological neighborhood, and the algorithm is such that neighboring neurons in the topological space tend to arrange themselves over time into a grid in feature space that mimics the neighborhood structure in the topological space. The SOFM algorithm differs from the Kohonen's neural networks SOM algorithm by dropping the topological neighborhood and replacing it with the concept of a global neighborhood generated by ranking with two significant variants. Whenever the SOFM and RMCT in the Consensus Networking machines gave conflicting decisions, we needed additional computational intelligence algorithms to break the tie. This motivated us to develop the Boosting with Bagging algorithm that is applied to SOFM, RMCT and PSHNN for the final majority voting decision. Boosting is a computational intelligence algorithm that can be combined with Bagging to improve the performance of a classifier. When combined appropriately, Boosting with Bagging is resistant to overfitting. While the original boosting algorithm is due to Schapire, later Freund and Schapire introduced an improved algorithm called Adaboost that was designed to handle 2-class classifiers. There were several extensions to the multiclass case, including Adaboost.M1. As we are interested in incorporating useful confidence information into a classifier, we combine bagging with a generalization of traditional boosting algorithm that allows confidence information to be incorporated. Our combined Boosting with Bagging algorithm emphasizes weaker learner for each boosting run.

Assuming we have N training instances, then we construct classifying function $f({\vec{x}}_{i})$. Class label y_i_ is either 0 or 1. The square error of classifier $f\left({\vec{x}}_{i}\right)$ is given by:

$${\left\{f\left({\vec{x}}_{i}\right)\;-y_{i}\right\}}^{2}$$

The procedure of Boosting with Bagging is described as following

• Initialization:

$$\alpha_{0}=\;1;t\;=\;1;\;W_{i}\;=\;p_{i}\;=\;1/N$$

where i = 1, 2, 3,…, N; N is the number of training instances;

W_i_ is the weight of training instance; P_i_ is the probability of instance.

• For t = 1 to T, take n subsamples, choose one of subsamples that gives smallest error.

$$\varepsilon_{t}\;=\;\sum_{i=1}^{N}P_{i}^{t}\left(1-h_{ty_{1}}\left(x_{i}\right)\right)$$

Update coefficient α*_t_*, weight W_i_ of training instance and probability P_i_ of instance at t boosting round.

$$\begin{array}{l} \alpha_{t}=\ln\left(-\frac{\varepsilon_{t}}{1-\varepsilon_{t}}\right)\\ W_{i}^{t+1}=W_{i}^{t}e^{-a_{t}}h_{y_{i}}^{t}\left({\vec{x}}_{i}\right)\\ P_{i}^{t+1}=\frac{W_{i}^{t+1}}{\sum_{i=1}^{N}W_{i}^{t+1}}\\ \text{t}=\text{t}+1; \end{array}$$

End

• The confidence instance $\vec{x}$ belonging? to class k is determined by the following equation: ${\overset{⌢}{\theta }}_{1},{\overset{⌢}{\theta }}_{2},{\overset{⌢}{\theta }}_{2},{\overset{⌢}{\theta }}_{3},\;........\;{\overset{⌢}{\theta }}_{n}$

Bagging with boosting will reduce variance error but will not affect bias error. It can be verified as following:

Assume that we want to form an estimator of a quantity based on observations. We can express the error of this estimate as the sum of a variance component and a bias component. Let us assume observations $x_{1},\;x_{2},\;x_{3},\;........\;x_{n}$ Estimator $\overset{⌢}{\theta }\left(x_{1},\;x_{2},\;x_{3},\;........\;x_{n}\right)$ and corresponding true $\theta \left(x_{1},\;x_{2},\;x_{3},\;........\;x_{n}\right)$. Thus

$$\begin{array}{ll} \text{Error} & =E{\left[{\left(\overset{⌢}{\theta }-\theta \right)}^{2}\right]}^{1}=E[{\left\{\overset{⌢}{\theta }-E(\overset{⌢}{\theta })+(E[\overset{⌢}{\theta }]-\theta )\right\}}^{2}]\\ & =E[{\left(E[\overset{⌢}{\theta }]-\overset{⌢}{\theta }\right)}^{2}+2(\overset{⌢}{\theta }-E(\overset{⌢}{\theta }))(E[\overset{⌢}{\theta }]-\theta )+{\left(E[\overset{⌢}{\theta }]-\theta \right)}^{2}]\\ & =E[{\left(E[\overset{⌢}{\theta }]-\overset{⌢}{\theta }\right)}^{2}]+E[2(\overset{⌢}{\theta }-E[\overset{⌢}{\theta }])(E[\overset{⌢}{\theta }]-\theta )+E[{\left(E[\overset{⌢}{\theta }]-\theta \right)}^{2}]\\ & =\text{Var}(\overset{⌢}{\theta })+{\left\{\text{Bias}(\overset{⌢}{\theta },\theta )\right\}}^{2} \end{array}$$

And there are m observed estimators: ${\overset{⌢}{\theta }}_{1},\;{\overset{⌢}{\theta }}_{2},\;{\overset{⌢}{\theta }}_{2},\;{\overset{⌢}{\theta }}_{3},\;........\;{\overset{⌢}{\theta }}_{n}$, average their predictions to obtain an overall estimation. The variance of the overall estimate is:

$$\begin{array}{l} Var\left(\overset{⌢}{\theta }\right)\;=\;Var\left(\frac{1}{m^{2}}\sum_{i=1}^{m}\overset{⌢}{\theta }\right)=\frac{1}{m^{2}}\left(Var\sum_{i=1}^{m}{\overset{⌢}{\theta }}_{i}\right)\\ \;\;\;\;\;\;\;\;\;=\;\frac{1}{m^{2}}\;\sum_{i=1}^{m}Var\left({\overset{⌢}{\theta }}_{i}\right)\;≅\;\frac{1}{m^{2}}\;mVar\left({\overset{⌢}{\theta }}_{i}\right)\;=\;\frac{1}{m}Var\left({\overset{⌢}{\theta }}_{i}\right) \end{array}$$

while the bias of the overall estimate is:

$$\begin{array}{l} Bias(\bar{\overset{⌢}{\theta }},\;\theta )\;=\;Bias\{\frac{1}{m}\;\sum_{i=1}^{m}{\overset{⌢}{\theta }}_{i}\;-\;\overset{⌢}{\theta }\}\\ \;\;\;\;\;\;\;\;\;\;\;\;\;\text{​}=\;Bias\{\frac{1}{m}\;\sum_{i=1}^{m}{\overset{⌢}{\theta }}_{i}\;-\;\frac{1}{m}\;\sum_{i=1}^{m}\theta \}\;=\;Bias\{\frac{1}{m}\sum_{i=1}^{m}\left({\overset{⌢}{\theta }}_{i}\;-\;\overset{⌢}{\theta })\right\}\\ \;\;\;\;\;\;\;\;\;\;\;\;\;=\;\frac{1}{m}\;\sum_{i=1}^{m}Bias\{{\overset{⌢}{\theta }}_{i},\;\theta \}\;≅\;\frac{1}{m}\;mBias({\overset{⌢}{\theta }}_{i}\;-\;\theta )\\ \;\;\;\;\;\;\;\;\;\;\;\;\;=\;Bias({\overset{⌢}{\theta }}_{i}\;-\;\theta ) \end{array}$$

Therefore, we can see that variance of the overall estimator is reduced, while the bias remains roughly the same.

### Improving the predicting power of computational intelligence by feature filtering and feature selection

In classification problems, we are often interested in maximizing the true positive rate (also called the sensitivity), as this rate reflects the ability of the classifier to detect the “signal”. For example, we designed this computational intelligence system classifier to indicate whether or not a given patient has malignant cancer (in this case the “signal” is “having malignant carcinoma”), then the cost of saying that the patient does not have malignant carcinoma when in fact the patient does (the false negative rate) is much higher than the cost of saying that the patient has malignant cancer when in fact the patient does not (the false positive rate). Thus, it is more important to make the false negative rate smaller and lower than the false positive rate. Since true positive rate = 1—false negative rate and true negative rate = 1—false positive rate, it is desirable in many applications to make the true positive rate (i.e. the sensitivity) larger at the expense of the true negative rate (i.e. the specificity). Sensitivity makes the y-axis and (1-specificity) makes the x-axis in Receiver Operating Characteristic (ROC) curve. A complete prefect random “classifier” gives a diagonal line with Youden Index = 0 (Youden index is the sensitivity + specificity—1), while a perfect deterministic classifier always gives both sensitivity and accuracy equal to 1 with Youden Index = 1. A large ROC area and a large Youden Index indicate a good classifier. In our case, a true positive corresponds to the case of correctly classifying a malignant cancer patient. Malignant cancers tend to be less distinctive than benign compare to normal tissues. Characteristic tumor associated gene expressions may turn out to have desirable properties that can be used to enhance sensitivity at the expense of specificity. To qualify for features (measured by experiments) in our classifier, any two features must not be statistically correlated, must give a satisfactory distance separation in the feature space (between classes) and must offer good generalization for the predictor [110-112].  The system we developed satisfies the above criteria and is a useful tool for enhancing accuracy upon diagnosing diseases and predicting prognosis.

## Competing interests

The authors declare that they have no competing interests.

## Authors' contributions

JYY, MQY and ZL conceived the project, designed the algorithms and experiments, and performed the study. YM and JL performed immunohistochemistry experiments. YD assisted the study. ZL and JY drafted the manuscript and XH finalized manuscript. ZL supervised the study.

**Figure 9:**
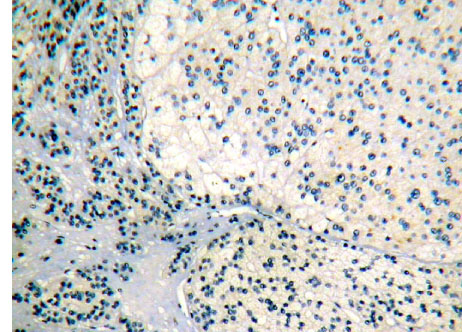
Adrenocortical hyperplasia PCNA(—) Superision^TM^
